# Susceptibility of bacterial etiological agents to commonly-used antimicrobial agents in children with sepsis at the Tamale Teaching Hospital

**DOI:** 10.1186/1471-2334-13-89

**Published:** 2013-02-18

**Authors:** Samuel EK Acquah, Lawrence Quaye, Kenneth Sagoe, Juventus B Ziem, Patricia I Bromberger, Anthony A Amponsem

**Affiliations:** 1 Tamale Teaching Hospital, Tamale, Ghana; 2 Department of Medical Laboratory Services, School of Medicine and Health Sciences University for Development Studies, Tamale, Ghana; 3 School of Medicine and Health Sciences University for Development Studies, Tamale, Ghana; 4 Department of Neonatology, Southern California Kaiser Permanente Medical Group, San Diego, CA, USA

## Abstract

**Background:**

Bloodstream infections in neonates and infants are life-threatening emergencies. Identification of the common bacteria causing such infections and their susceptibility patterns will provide necessary information for timely intervention. This study is aimed at determining the susceptibilities of bacterial etiological agents to commonly-used antimicrobial agents for empirical treatment of suspected bacterial septicaemia in children.

**Methods:**

This is a hospital based retrospective analysis of blood cultures from infants to children up to 14 years of age with preliminary diagnosis of sepsis and admitted to the Neonatal Intensive Care Unit (NICU) and Paediatric Wards of the Teaching Hospital Tamale from July 2011 to January 2012.

**Results:**

Out of 331 blood specimens cultured, the prevalence of confirmed bacterial sepsis was 25.9% (86/331). Point prevalence for confirmed cases from NICU was 44.4% (28/63) and 21.6% (58/268) from the Paediatric ward. Gram positive cocci (GPC) were the predominant isolates with Coagulase positive (32.2%) and Coagulase-negative (28.7%) *Staphylococci* accounting for 60.9% of the total isolates. Gram negative rods (GNR) comprised 39.1% of all isolates with *Klebsiella, E*.*coli* and *Salmonella* being the most common organisms isolated. *Klebsiella* was the most frequent GNR from the NICU and *Salmonella typhi* was predominantly isolated from the paediatric ward. *Acinetobacter* showed 100.0% susceptibility to Ceftriaxone and Cefotaxime but was resistant (100.0%) to Ampicillin, Tetracycline and Cotrimoxazole. *Escherichia coli* and *Klebsiella* were 80.0% and 91.0% susceptible to Ceftriaxone and Cefotaxime respectively. *Klebsiella* species showed 8.3% susceptibility to Tetracycline but was resistant to Ampicillin and Cotrimoxazole. *Escherichia coli* showed 40.0% susceptibility to Ampicillin, Chloramphenicol and Cotrimoxazole; 20.0% susceptibility to Tetracycline and 80.0% susceptible to Gentamicin and Cefuroxime. Coagulase negative *Staphylococci* was susceptible to Gentamicin (72.0%) but Coagulase positive *Staphylococci* showed intermediate sensitivity to Gentamicin (42.9%).

**Conclusion:**

Coagulase Negative, Coagulase Positive *Staphylococci, Salmonella* and *Klebsiella* were the aetiological agents of bloodstream infection among children at TTH. While gram-positive and gram-negative bacteria showed low susceptibility to Ampicillin, Tetracycline and Cotrimoxazole, the GNR were susceptible to Gentamicin and third-generation cephalosporins.

## Background

The World Health Organization (WHO) reported in 2005 that over 70 percent of deaths in children under age five occur within the first year of life and 40 percent occur within the first month of life [1]. The causes of death according to the report were attributable to malnutrition and infectious diseases [1]. In Ghana the estimated number of deaths among children aged 1-59 months was 32,052, while 22,672 deaths were estimated to have occurred among children 0-27 days, with neonatal sepsis accounting for 4,923 deaths in 2008 [2]. Sepsis is a blood stream infection usually caused by pathogenic bacteria with the diagnosis often beginning with clinical suspicion [3]. However, in children the symptoms are often non-specific, and the clinical course may be fulminant, quickly progressing to a medical emergency that requires urgent attention [4,5]. The principal method of diagnosing sepsis is the isolation of causative organisms from blood cultures [6]. In industrialized countries Group B *Streptococcus* (GBS) and *Escherichia coli* have been reported as the most frequent bacterial etiological agents of neonatal and infant sepsis [7,8]. While *Escherichia coli* and other gram-negative rods (GNR) have been reported to be responsible for the high morbidity and mortality rates among children in some developing countries [9,10], sepsis among Ghanaian children is predominantly caused by *Staphylococcus aureus* and non-typhoid *Salmonellae*[11,12]. Blood culture and antimicrobial susceptibility testing results are usually available between 48-72 hours after the specimen is obtained and therefore initial antimicrobial treatment has been usually empirical with the aim that the most likely pathogens would be susceptible to the chosen drugs [5]. However, this initiative is undermined by the fact that the spectrum of bacteria and their susceptibility patterns may vary over time, depending on the prevailing conditions such as patient population, antimicrobial drug usage and healthcare worker’s infection control practices. If not monitored, this could lead to inappropriate use of antibiotics and a subsequent increase in antimicrobial resistant organisms [13-15]. In order to guide empirical therapy, this study reports on bacterial isolates and their susceptibility to commonly-used antimicrobial agents in the empirical treatment of suspected bacterial septicaemia in children presenting at the Tamale Teaching Hospital.

## Methods

### Study period/site

This retrospective study was conducted at the Tamale Teaching Hospital (TTH) from July 2011 to January 2012. TTH is a 340 bed complement hospital situated in the Northern Region of Ghana. In addition to offering clinical care to the inhabitants of Tamale and its surrounding environ, it also serves as a referral hospital to the two Upper regions (Upper East and Upper West) of Ghana. The hospital runs six clinical departments including Paediatrics. With a bed complement of 60, the Paediatric ward receives admissions from the Children’s Emergency Ward (CEW), reporting cases from home or as referrals from other hospitals. The CEW serves as an outpatient clinic to the surrounding population and sees patients up to 14 years of age. The ward has 11 beds for emergency stabilization and holds children for up to 24 hours for treatment with onward admission to the Pediatric ward when hospitalization becomes necessary. All infants less than 30 days of age are admitted to the Neonatal Intensive Care Unit (NICU) which has a bed complement of 17. TTH is an affiliated teaching hospital for the School of Medicine and Health Sciences, University for Development Studies (UDS). Approval for the study was obtained from the Research and Planning Unit for Ethics of TTH.

### Data extraction

Data comprising age, sex, bacterial isolates and their susceptibility reports recorded from July 2011 to January 2012 were extracted from the blood culture entry books of the Bacteriology unit of the hospital’s Laboratory Department. All cases of blood culture requested with a preliminary diagnosis of sepsis from a newborn up to 14 years of age admitted at the NICU, children’s emergency and the pediatric wards were included in the study.

### Laboratory procedures

#### Culture

Blood specimens were taken into BD Ped culture broth bottles (BD 7 Loveton Circle, Sparks Maryland USA) after thorough cleaning of the venipuncture site and the rubber cap of the bottle with 70% alcohol. The culture bottles were then incubated at 35°C in the BACTEC® 9050 blood culture system (BD 7 Loveton Circle, Sparks Maryland USA) for 5 days after which negative indicating specimen are discarded [16,17]. Positive indicated culture bottles were removed and 1 ml of broth aseptically withdrawn from the culture bottles using a sterile syringe and needle. Two drops of the broth were inoculated onto MacConkey Agar, Blood Agar (BA) and Chocolate Agar (CA) plates respectively. The BA and CA plates were placed in candle extinction jars and incubated at 37°C for 18 to 24 hours together with the MacConkey plates.

### Gram stain

Gram staining for bacterial identification was performed on heat-fixed smears prepared from the remaining broth following standard bacteriological protocol described by [18].

### Bacterial identification

Only plates that showed homogenous colony appearance were followed-up for bacterial identification. Conventional identification methods based on characteristics such as morphology, appearance in culture media, physiological and biochemical properties were relied upon to properly identify isolated organisms [18]. The BBL Crystal Auto reader (BD 7 Loveton Circle, Sparks Maryland USA) was employed where conventional methods for identification were inconclusive. Tube coagulase was performed for isolates of gram positive cocci in clusters [18,19]. Antimicrobial susceptibility tests were done using the Kirby-Bauer disk diffusion method [20] and interpreted according to the Clinical and Laboratory Standard Institute (CLSI) criteria. Susceptibility testing was controlled with American Type Culture Collection (ATCC) strains: *Staphylococcus aureus* ATCC 25923, *Escherichia coli* ATCC 25922 and *Pseudomonas aeruginosa* ATCC 27853 [21].

### Data analysis

Data retrieved were entered into Microsoft Excel and analyzed using GraphPad Prism® Version 5.0 for Windows (GraphPad Software, San Diego, CA, USA). Descriptive statistics was employed to explain the general distribution of data. Categorical variables were compared using Chi-square test where appropriate. For all statistical comparisons a *p*-value of <0.05 was considered statistically significant.

## Results

### Study population

During the study period, spanning July 2011 to January 2012, NICU registered a total of 1074 admissions. The paediatric ward registered a total of 3110 admissions, of which 2537 were received from the Children’s Emergency Ward (Institutional care report 2012). The median age of infants with positive blood culture in NICU was 5 days with that for children being 3 years.

### Blood cultures

During the study period, a total of 331 blood cultures with complete demographic and bacteriological data were received. Two hundred and sixty-eight (81.0%) were from the paediatric ward and 63 (19%) were from NICU. Out of the 331 blood cultures, 97 (29.3%) were flagged positive. Ten (10) of the positively flagged samples were considered as being contaminated upon the isolation of *Corynebacterium* species, *Bacillus* species and *Micrococcus* species which were considered as contaminants [22,23]. One positive sample vial was indicative of the presence of yeast leaving 86 true positive (pure culture) samples to be followed. The estimated overall contamination rate was 3% with confirmed bacterial sepsis rate being 26.0% (86/331).

### Positive bacterial isolates stratified by ward

Of the 63 cultures submitted from the NICU, 28 (44%) were positive out of which 15 (35%) cases were early onset infections (less than 7 days of age) and 13 (42.8%) cases were late onset infections (greater than 7 days of age.) Of the 268 cultures submitted from the paediatric ward, 58 (21.6%) were positive for bacterial pathogens.

Upon stratification into their gram reactions, 55 (64.0%) were gram-positive cocci (GPC) and 31(36.0%) were gram-negative rods (GNR). Coagulase positive and Coagulase-negative *Staphylococci* were the predominant GPC isolates recovered from all the wards, accounting for 32.2% and 28.7% of the total isolates respectively. For the GNR’s, *Klebsiella* was the most frequent isolate among the neonates and children from the paediatric ward. *Escherichia coli* were recovered in similar proportions across the wards with *Salmonella typhi* being the major cause of bacterial sepsis in children admitted to the paediatric ward. *Streptococci* and *Acinetobacter* species were only rarely recovered from NICU and the paediatric ward respectively (Table 1).

**Table 1:** Bacterial pathogens isolated from the two wards

	**NICU**		**PAED**	**TOTAL**
	**(N = 63)**		**(N = 268)**	**(N = 331)**
**Pathogens**	**EOS(%)**	**LOS(%)**	**n(%)**	**n(%)**
**Gram positive cocci (GPC)**				
*Coagulase Positive Staphylococci*	3(20.0)	5(38.5)	20(34.5)	28(32.2)
*Coagulase Negative Staphylococci*	5(33.3)	5(38.5)	15(25.9)	25(28.7)
*Streptococcus pneumoniae*	0(0.0)	0(0.0)	1(1.7)	1(1.2)
*β-haemolytic Streptococcus*	0(0.0)	0(0.0)	1(1.7)	1(1.2)
**Gram negative rods (GNR)**				
*Klebsiella pneumoniae*	2(13.3)	0(0.0)	4(6.9)	6(6.9)
*Escherichia coli*	1(6.7)	1(7.7)	4(6.9)	6(6.9)
*Salmonella typhi*	0(0.0)	0(0.0)	6(10.3)	6(6.9)
*Klebsiella spp*	2(13.3)	0(0.0)	4(6.9)	6(6.9)
*Pseudomonas aeruginosa*	0(0.0)	1(7.7)	2(3.4)	3(3.5)
*Non-typhoidal Salmonella*	1(6.7)	0(0.0)	1(1.7)	2(2.3)
*Acinetobacter spp*	1(6.7)	1(7.7)	0(0.0)	2(2.3)
**Total isolates**	**15**	**13**	**58**	**86**

NICU: Neonatal intensive care unit, PAED: Paediatric ward, EOS: Early onset, LOS Late onset.

### Antibiotic sensitivity profiles of isolated bacterial pathogens

Table 2 shows the susceptibilities of the isolated pathogens from the two units. Among the antimicrobials used, third-generation cephalosporins (Ceftriaxone and Cefotaxime) showed good activity against the GNR isolates. *Salmonella* and *Acinetobacter* showed 100.0% susceptibility, while *Escherichia coli* and *Klebsiella* were 80.0% and 91.0% susceptible to Ceftriaxone and Cefotaxime respectively. *Salmonella* and *Acinetobacter* were 100.0% resistant to Ampicillin, Tetracycline and Cotrimoxazole. *Klebsiella* species showed 8.3% susceptibility to Tetracycline but were 100.0% resistant to Ampicillin and Cotrimoxazole. *Escherichia coli* showed variable susceptibility to the tested antimicrobials with 40.0% susceptible to Ampicillin, Chloramphenicol and Cotrimoxazole; 20.0% susceptible to Tetracycline; and 80.0% susceptible to Gentamicin and Cefuroxime. The susceptibility of *Pseudomonas aeruginosa* to the commonly used antimicrobials, Gentamicin, Ceftriaxone, and Cefotaxime, is as shown in Table 2. Levels of susceptibility of the GPC to the available antimicrobials also varied greatly. Most Coagulase positive *Staphylococci* (96.4%) and the Coagulase negative *Staphylococci* (96.0%) were resistant to Ampicillin and Penicillin. Coagulase negative *Staphylococci* was often sensitive to Gentamicin (72.0%) but Coagulase positive *Staphylococci* had only intermediate sensitivity to Gentamicin (42.9%) (Table 2).

**Table 2:** Bacterial isolates and susceptibility patterns among study population

		**GEN**	**CRX**	**CHLO**	**CTR**	**CTX**	**AMP**	**TET**	**COT**	**PEN**	**FLX**	**ERY**
**Bacteria isolates**	**N**	**n(%)**	**n(%)**	**n(%)**	**n(%)**	**n(%)**	**n(%)**	**n(%)**	**n(%)**	**n(%)**	**n(%)**	**n(%)**
*Salmonella spp*	**8**	-	-	5(62.5)	8(100.0)	8(100.0)	0(0.0)	0(0.0)	0(0.0)	-	-	-
*Klebsiella spp*	**12**	4(33.3)	1(8.3)	5(41.7)	11(91.7)	11(91.7)	0(0.0)	1(8.3)	0(0.0)	-	-	-
*Escherichia coli*	**6**	4(66.7)	4(66.7)	2(33.3)	4(66.7)	4(66.7)	2(33.3)	1(16.7)	2(33.3)	-	-	-
*Acinetobacter spp*	**2**	2(100.0)	0(0.0)	0(0.0)	2(100)	2(100.0)	0(0.0)	0(0.0)	0(0.0)	-	-	-
*Pseudomonas aeruginosa*	**3**	1(33.3)	-	-	1(33.3)	1(33.3)	-	-	-	-	-	-
*Coagulase negative Staphylococci*	**25**	18(72.0)	14(56.0)	-	-	-	1(4.0)	6(24.0)	5(20.0)	1(4.0)	10(40.0)	13(52.0)
*Coagulase positive Staphylococci*	**28**	12(42.9)	16(57.1)	-	-	-	1(3.6)	2(25)	2(7.1)	1(3.6)	8(28.6)	10(35.7)

GEN –Gentamicin, CRX-Cefuroxime, CHLO-Chloramphenicol, CTR-Ceftriaxone, CTX- Cefotaxime, AMP-Ampicillin, TET- Tetracycline, COT-Cotrimoxazole, PEN- Penicillin, FLX-Flucloxacillin, ERY- Erythromycin, (-) – Not done, Salmonella spp= Salmonella spp and Salmonella typhi, Klebsiella spp = Klebsiella spp and Klebsiella pneumoniae.

## Discussion

Bloodstream infection remains a major cause of morbidity and prolonged hospital stay in children [24]. In neonates and infants, it is a life-threatening emergency, and therefore identifying the common bacteria and their susceptibility patterns will provide information necessary for timely intervention [25,26]. Unfortunately there is limited information about the spectrum of pathogens causing infection and their antibiotic susceptibility in many under-resourced areas [27]. Over the six months study period, this study showed a 25.9% positivity rate for isolated bacterial pathogens. This could most likely represent an under-estimation of the true prevalence of bacterial septicaemia as many of the infants and children cared for in NICU and the paediatric ward respectively received empirical treatment with antibiotics before blood was drawn for culture. Polage *et al.*[28] iterated this fact in their study on laboratory use in Ghana where they concluded that physician perception regarding the value of diagnostic testing has been a major potential barrier to laboratory use resulting in empiricism and disproportionate antimicrobial administration. The estimated positivity rate for isolated bacterial pathogens from this study is however lower than rates found in University of Calabar Teaching Hospital (48.9%) and University of Port Harcourt Teaching Hospital (34.2%) in Nigeria [29,30].

The spectrum of gram-negative bacteria responsible for neonatal sepsis in this study is similar to that reported by other authors [31-33] with the predominant isolates being *Klebsiella* and *Escherichia coli*. *Klebsiella* may be regarded as normal flora in many parts of the body. They colonize the skin, pharynx, and the gastrointestinal tract. They may, however, be associated with chorioamnionitis, urogenital infection or urinary tract infection in the mother. Neonates could therefore acquire these organisms before and/or during delivery from their mothers with infections at the time of delivery or shortly thereafter [34].

As reported in other studies [35,36], the GPC were the predominant bacteria isolates in both early and late onset sepsis from NICU. The high proportions of CoNS isolated in both early onset (33.3%) and late onset (38.5%) at the NICU gives a suggestion of CoNS as an emerging pathogen in the unit [37]. However, the diagnosis of CoNS in bloodstream infections is problematic. Generally, CoNS is not thought to be a cause of EOS, although it has been reported in studies reviewing organisms causing early infections [38,39]. Conversely, CoNS is the predominant cause of LOS in low birth weight infants and infants who have indwelling catheters or other instrumentation [40,41]. The isolation of CoNS in blood cultures is considered to be pathogenic if it is obtained from at least two separate sites, a process which may be difficult in infants [40]. Therefore the combination of isolation of CoNS from the bloodstream with clinical symptoms of neonatal infection may be the best approach for determining pathogenicity of the CoNS [41,42]. Since CoNS is the predominant bacteria colonizing the skin, it is very easy to contaminate the blood culture during the process of venipuncture. The optimal technique for obtaining blood cultures specifies povidone-iodine or chlorhexidine as skin-cleansing agents and venipuncture techniques that minimize contamination by skin flora [41]. Generally, 70% alcohol serves as the major cleansing agent of the skin in resource-limited areas in view of perennial unavailability of povidone and chlorhexidine in both the NICU and paediatric wards. These factors suggest that the high proportion of the CoNS isolated is more likely to be contamination from the skin [26,43].

Consistent with other studies [44,45], *Streptococcal* species were recovered infrequently from neonates with early onset (EOS) in this study. There is an on-going debate about the importance of GBS infections in sub-Saharan Africa [46,47]. Although most studies report that GBS infections are rare in Africa, studies in Malawi, Kenya, South Africa and Zimbabwe report that maternal colonization and EOS infections rates are similar to those reported from the United States and Europe [48]. It has been hypothesized that the overwhelming nature of the infection in the neonate with early death, a predominantly out-born population, the infrequency of obtaining blood cultures in sick infants, and the fastidious culture requirements to identify GBS might interfere with its identification as a major pathogen in the neonatal period [48]. Other authors suggest that the cause of EOS is gradually shifting from vertical transmission to acquisition of infection from immediate exposure to environment such as hands of health-care workers or unclean delivery practices [44,45]. The yield of GBS from blood cultures is greatly influenced by many factors such as methodology of obtaining blood cultures, recent or intrapartum antibiotic use, and requirement for specialized media [49]. Interpretation of results from this study should be done cautiously as most of these factors were not factored into the inclusion criteria per the design of the study.

In spite of the fact that late-onset (LOS) neonatal infection may occur through vertical transmission during passage through the birth canal leading to colonization and, later, to infection [50], the majority of the LOS in newborn infants is usually hospital-acquired infection [26]. Although some of the blood cultures from babies greater than seven days of age were obtained from infants who had been admitted to the NICU immediately after birth and then became sick (representing hospital-acquired infection), many of the babies who had blood cultures drawn in the NICU were admitted directly from home or from the outpatient department after seven days of age. Infants, less than 30 days of age are also admitted to the NICU rather than the paediatric ward. Isolates from these admissions therefore, are more likely to represent community-acquired rather than hospital- associated late onset infections.

Other studies have reported non-typhoid *Salmonella* as the most frequent cause of bloodstream infection in older children [11,51,52] however the predominant *Salmonella* species isolated from the paediatric wards in this study was *Salmonella typhi*. It has been suggested that in addition to poor personal hygiene of caretakers and environmental contaminations, young children are more susceptible to *Salmonella* infection because they possess immature gut lymphoid tissues and have decreased gastric acidity [11,53]. This finding underscores the need for increased awareness and knowledge about cleanliness and environmental sanitation for healthy living in the communities.

As observed from the current study, both the gram-positive (4% - 25%) and gram–negative (0% - 40%) bacteria showed very low sensitivity to Ampicillin and Cotrimoxazole. However the gram negative bacteria displayed high levels of susceptibility to the third-generation cephalosporins with the exception of *Pseudomonas aeruginosa*. Gentamicin was effective against *Escherichia coli* (80%), *Acinetobacter* (100%) and the CoNS (72%), but *Klebsiella* (33.3%), *Pseudomonas* (33.3%) and Coagulase positive *Staphylococci* (42.9%) were often resistant to Gentamicin.

The recommended empiric antibiotic therapy for EOS has been Ampicillin and an aminoglycoside, usually Gentamicin, while in LOS a third-generation cephalosporin in combination with Ampicillin is used [54,55]. The noteworthy findings of this study are the susceptibilities of the GNR to Gentamicin and third-generation cephalosporins. Many authors report increasing GNR resistance to both Gentamicin and third-generation cephalosporins in developing countries which may be related to frequent and unrestricted use of third-generation cephalosporins [56-59]. It has been suggested that over-use of third-generation cephalosporins is also linked to increased risk of neonatal death [60], increased risk for necrotizing enterocolitis in low birth-weight infants [61] and increased risk for invasive candidiasis [62]. Of particular interest are some *Acinetobacter* species that have emerged in Southeast Asia and Africa that are multi-drug resistant [63,64]. Results from this study shows that the isolated GNRs are still fairly susceptible to third-generation cephalosporins. First-line empiric therapy for rule-out sepsis at the NICU in TTH currently utilizes a combination drug comprising Ampicillin and Gentamicin while the third-generation cephalosporins are only used very selectively, a practice that may reduce risk for multi-drug resistant bacterial strains.

## Conclusions

This review identified Coagulase Negative, Coagulase Positive *Staphylococci, Salmonella* and *Klebsiella* as predominant aetiological agents of bloodstream infection among children at TTH. While both the gram-positive and gram- negative bacteria showed very low susceptibility to Ampicillin, Tetracycline and Cotrimoxazole, the GNR were fairly susceptible to Gentamicin and third-generation cephalosporins. Further studies to identify risk factors for developing sepsis and designing interventions to reduce bloodstream infections in the wards and the communities should be piloted.

### Limitations

Factors such as recent or intrapartum antibiotic use and requirement for specialized media were not factored into the inclusion criteria per the design of the study. Data for this study were obtained over the course of seven months between July 2011 and January 2012, and therefore seasonal variations in the frequency of bloodstream infection and causative microorganisms could not be assessed.

## Competing interests

We declare that we have no competing interests.

## Authors’ contributions

SEKA carried out the cultures, designed and drafted the manuscript, LQ performed the statistical analysis and participated in the drafting of the manuscript, PIB, KS, JBZ and AAB coordinated the study and participated in the drafting of the manuscript. All authors read and approved the final manuscript.

## Pre-publication history

The pre-publication history for this paper can be accessed here:

http://www.biomedcentral.com/1471-2334/13/89/prepub
